# Sensing small interaction forces through proprioception

**DOI:** 10.1038/s41598-021-01112-w

**Published:** 2021-11-08

**Authors:** Fazlur Rashid, Devin Burns, Yun Seong Song

**Affiliations:** 1 Department of Mechanical and Aerospace Engineering, Missouri University of Science and Technology, Rolla, MO 65401 USA; 2 Department of Psychological Science, Missouri University of Science and Technology, Rolla, MO 65401 USA

**Keywords:** Neuroscience, Engineering

## Abstract

Understanding the human motor control strategy during physical interaction tasks is crucial for developing future robots for physical human–robot interaction (pHRI). In physical human–human interaction (pHHI), small interaction forces are known to convey their intent between the partners for effective motor communication. The aim of this work is to investigate what affects the human’s sensitivity to the externally applied interaction forces. The hypothesis is that one way the small interaction forces are sensed is through the movement of the arm and the resulting proprioceptive signals. A pHRI setup was used to provide small interaction forces to the hand of seated participants in one of four directions, while the participants were asked to identify the direction of the push while blindfolded. The result shows that participants’ ability to correctly report the direction of the interaction force was lower with low interaction force as well as with high muscle contraction. The sensitivity to the interaction force direction increased with the radial displacement of the participant’s hand from the initial position: the further they moved the more correct their responses were. It was also observed that the estimated stiffness of the arm varies with the level of muscle contraction and robot interaction force.

## Introduction

Beyond traditional robots that perform isolated tasks away from human operators^1–4^ , future robots are expected to be physically closer to the users and perform interactive tasks^5–8^. In particular, robots that can physically interact with humans through direct contact have the potential to assist the human workforce in various scenarios, such as in healthcare, manufacturing, or education^9–12^. For example, the foreseen shortage of physical therapists and nurses amplifies the necessity for the development of effective and intuitive physical Human–Robot Interaction (pHRI). Robots may provide physical assistance to patients like human therapists would for effective movement assistance and rehabilitation^10–15^.

In order to advance pHRI, however, it is crucial to first understand the underlying mechanism of effective physical interaction from the perspective of human users^16^.Indeed, humans are experts of physical human–human interaction (pHHI) such as while hand-shaking^17,18^, walking together^16,19,20^, or jointly carrying loads^21,22^. In many pHHI tasks, humans coordinate their movements together, not through verbal communication or visual feedback, but through the interaction forces acting at their arms and hands^20^. This physical communication between partners can lead to improved performance in the absence of explicitly shared motor goals^19,23–27^, a distinction of skill levels^20^ or roles^28^, or even motor adaptation^29–31^. These information-carrying interaction forces are typically 20 N or less^20,32^, are usually kept below 10 N^33,34^, and can sometimes be as low as 1 N^35^. It would then be required of the humans in physical interaction tasks to be sensitive to the small changes in the interaction forces for better motor communication with the partner.

How, then, do human partners remain sensitive to small interaction forces during physical interaction tasks? One possibility is through the mechanoreceptors distributed at the site of the physical coupling, typically through holding of hands^16,19,23,25^. However, these receptors may not be suitable for detecting subtle changes in the interaction forces due to the high preload of grip forces that is crucial to maintain a stable physical coupling^36,37^. That is, the small changes in the interaction forces could be below the Weber fraction (< 10 %) of the pre-existing stimuli on the pressor receptors (grip force), making them unreliable for detecting interaction forces^38,39^. If they relied only upon these mechanoreceptors, humans would have to loosen their grip for reliable motor communication at the cost of unreliable physical coupling.

Alternatively, proprioceptors on muscles, tendons, and joints may help detect the small interaction forces through the resulting kinematic displacements of the arm^40,41^.The interaction force at the hand will create the corresponding movement of the upper and lower arm, which then creates length changes in the muscles and tendons that are sensed by muscle spindles and/or Golgi tendon organs (GTOs)^42,43^. Even in certain interaction tasks where there is little arm movement (such as in^20^), small movements can create muscle length changes above the Weber fraction. In this view, small changes in the interaction forces may be detected by the proprioceptors, as long as the arm stiffness is low enough to allow detectable movement in response to the small interaction force.

To this end, this work is aimed at investigating the physical interaction strategy in humans for effective motor communication through small interaction forces. In particular, this work investigates the effect of the state of the arm in the sensitivity to the information provided by the interaction force from an external source. The hypothesis is that humans are more sensitive to the direction of the subtle push on their palm when the arm is displaced more as a consequence of the push. Supporting observations may indicate the presence of a specific pHHI/pHRI strategy that the humans modulate their arm stiffness to improve sensitivity to small interaction forces.

## Results

### Sensitivity to interaction forces is affected by the robot force as well as muscle contraction levels

Out of a total of 1920 trials among 20 participants, the number of correct responses was 1443 and the number of incorrect responses was 477 trials (including the number of no-response: 255).

**Figure 1:**
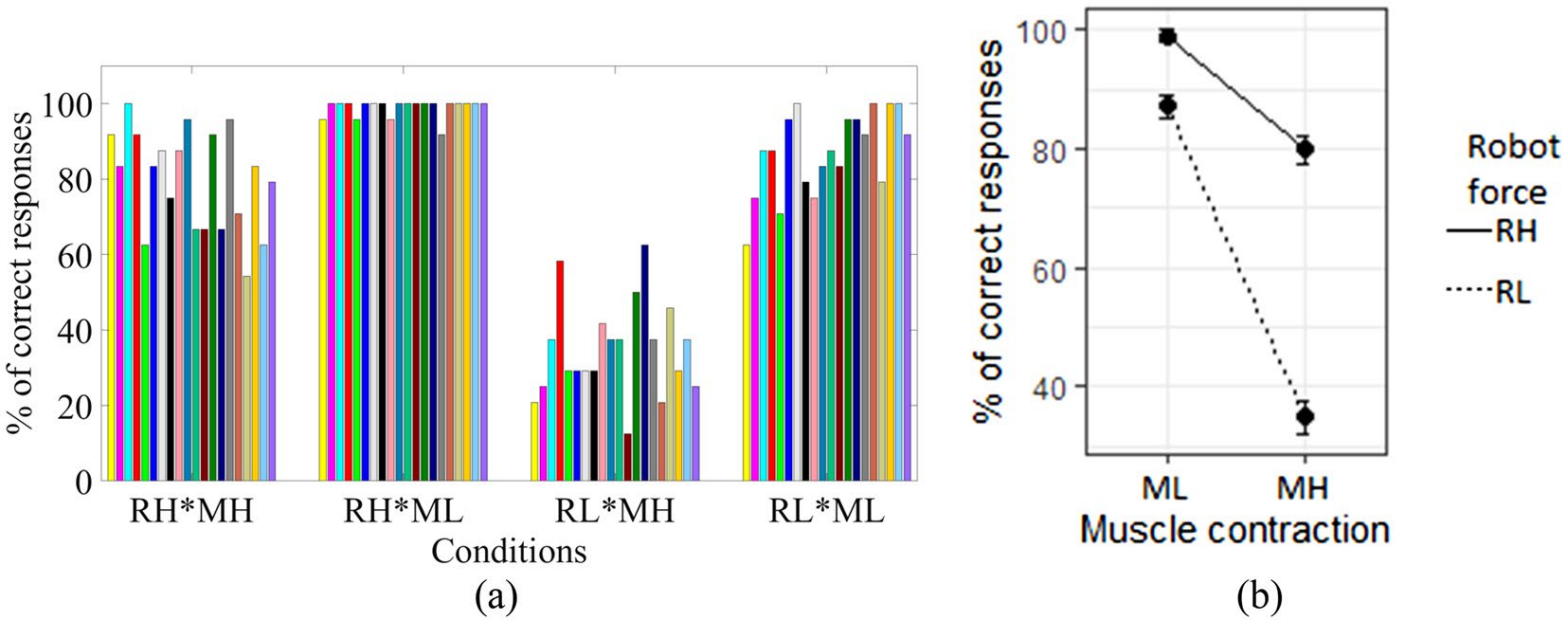
Percentage of correct responses varies with the level of muscle contraction where correctness was maximum (~ 100%) at high robot force with low muscle contraction (RH*ML) condition (**a**) experimental results (different colors represent different participants) (**b**) ANOVA analysis of the percentage of correct responses.

Among the 4 possible combinations of conditions, the sensitivity to the interaction force direction was highest when the applied force was high and the muscle contraction was low (RH*ML, average 99.0%), and lowest in RL*MH (average 34.8%, Fig. 1a). It was observed that with a low level of robot interaction force and low level of muscle contraction (RL*ML) condition, the percentage of correct responses was comparable (average 87.1%) with high robot interaction force and high muscle contraction (RH*MH, average 79.8%) condition, where in both RL*ML and RH*MH, the sensitivity was higher than in RL*MH and lower than in RH*ML. These trends were statistically significant where applying lower robot interaction force (RL*MH and RL*ML conditions) decreased the sensitivity by 11.88% (*p* < 0.001), whereas high muscle contraction (RH*MH and RL*MH conditions) decreased the sensitivity by 19.17% (*p* < 0.001, Fig. 1b). The combined effect of the low robot interaction force (RL) and the high muscle contraction (MH) was also significant, decreasing the sensitivity of the interaction force by an additional 33.13% (*p* < 0.001). These results are summarized in Table 1.

**Table 1:** Fixed effects of the percentage of correct responses using linear mixed model fit by REML and *t* tests use Satterthwaite's method (Response ~ Robot.force*Muscle.contraction + (1 | Participants)).

	Estimate	Std. Error	df (degree of freedom)	t value	Pr(>|t|)
Intercept	0.9896	0.02	102.92	45.51	*p* < 0.001***
RL	− 0.1188	0.03	137.00	− 4.17	*p* < 0.001***
MH	− 0.1917	0.03	137.00	− 6.73	*p* < 0.001***
RL*MH	− 0.3313	0.04	137.00	− 8.23	*p* < 0.001***

*p < 0.05, **p < 0.01, ***p < 0.001.

### High radial displacement of the hand increases the sensitivity to small interaction forces

**Figure 2:**
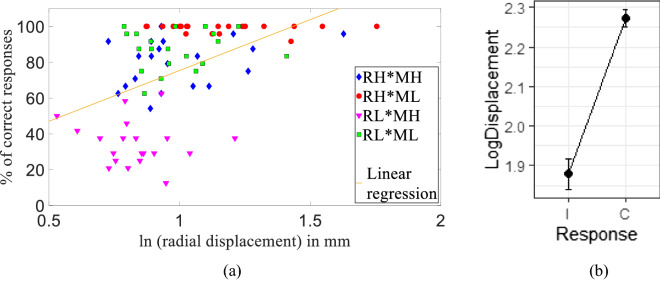
(**a**) Percentage of correct responses increases with radial displacement from the initial position and was highest during high robot interaction force with low muscle contraction (RH*ML, red) condition, during which the radial displacement was also high. Linear regression fit gives R^2^  =  0.228 (**b**) mean and standard deviation of logarithmic radial displacement of all correct response trials (**c**) was higher than incorrect response trials (I) (*p* < 0.001).

The radial displacement of the hand from the center (initial position) in each trial was strongly correlated with the sensitivity of the interaction force direction (Fig. 2a). The radial displacement was the highest during the RH*ML condition (red), during which the chance to make correct responses was also the highest. As the radial displacements are lower in RL*ML and RH*MH trials, the chance of correct responses was also lower. The radial displacement was the smallest in the RL*MH condition where the least correct responses were made. Linear regression showed a correlation of R^2^ = 0.228 between the percentage of correct responses and the radial displacement in a logarithmic scale. In addition, the radial displacement was higher in trials with correct responses than in trials with incorrect responses after removing participant variability (*p* < 0.001, Fig. 2b). Including participant variability, the logarithmic radial displacement of all trials with correct and incorrect responses was 2.270 ± 0.430 mm (out of 1443 trials) and 1.876 ± 0.302 mm (out of 477 trials), respectively. Paired sample *t* test showed a difference of 0.40 ± 0.26 mm with a large effect size (Cohen’s D = 1.54).

Then, a generalized linear mixed-effects model was used to analyze the items that affect the correctness of the response in a trial-by-trial manner, where the binomial outcome measure was whether participants responded correctly on that particular trial. These tests were conducted using the "lmerTest'' package for R.^44^ .We included fixed effects for the robot interaction force (two levels), muscle contraction (two levels), the direction of the push from the robot (one variable for X/Z and another for +/−), and logarithmic radial displacement on that particular trial, with a random intercept for participants (allowing for different overall sensitivity) and by-participant random slopes for muscle contraction (allowing the effect of contraction to differ across participants). Table 2 shows that the sensitivity to the direction of the force was reduced by low robot interaction force (RL, *p* < 0.001), high muscle contraction (MH, *p* < 0.001), and with Z-direction movement (DZ, *p* = 0.04), as indicated by the negative estimates and the corresponding odds ratios below 1 (where {odd ratio} = exp (estimate)). The participants were only 12% as likely to be correct in RL trials than in RH trials, only 6% as likely to be correct in MH trials than in ML trials, and only 77% as likely to be correct in the Z direction movements than in the X direction. On the other hand, the sensitivity was increased by positive direction pushes (Dir+, in X+ or Z+ directions, *p* < 0.001) and larger radial displacement (LogD, p=0.02), as indicated by the positive estimates and the corresponding odds ratios above 1. The participants were 1.68 times more likely to be correct in X+ or Z+ directions than in X− or Z− directions, and were 1.31 times more likely to be correct for every unit increase (1 log (mm)) in the logarithmic radial displacement. The number of observations in this analysis was 1920 (20 participants x 96 trials).

**Table 2:** Fixed effects of all parameters using generalized linear mixed model fit by maximum likelihood in a trial-by-trial manner (Response ~ Robot.force + Muscle.contraction + Direction + Dimension + LogDisplacement + (Muscle.contraction | Participant)).

	Estimate	Std. Error	z value	Odds ratio	Pr(>|z|)
(Intercept)	3.65	0.42	8.76		*p* < 0.001***
RL	− 2.10	0.15	− 14.06	0.12	*p* < 0.001***
MH	− 2.85	0.30	− 9.54	0.06	*p* < 0.001***
Dir+	0.52	0.13	3.91	1.68	*p* < 0.001***
DZ	− 0.27	0.13	− 2.03	0.77	0.04*
LogD	0.27	0.11	2.36	1.31	0.02*

*p < 0.05, **p < 0.01, ***p < 0.001.

Unlike the ANOVA analysis presented in Table 1, the interaction between the robot force and the muscle contraction was not significant in this analysis (and thus excluded from the final model), suggesting that it may have been a statistical artifact caused by the ceiling effect of near-perfect accuracy in the RH*ML condition.

### Estimated arm stiffnesses depend on both robot force and muscle contraction levels

**Figure 3:**
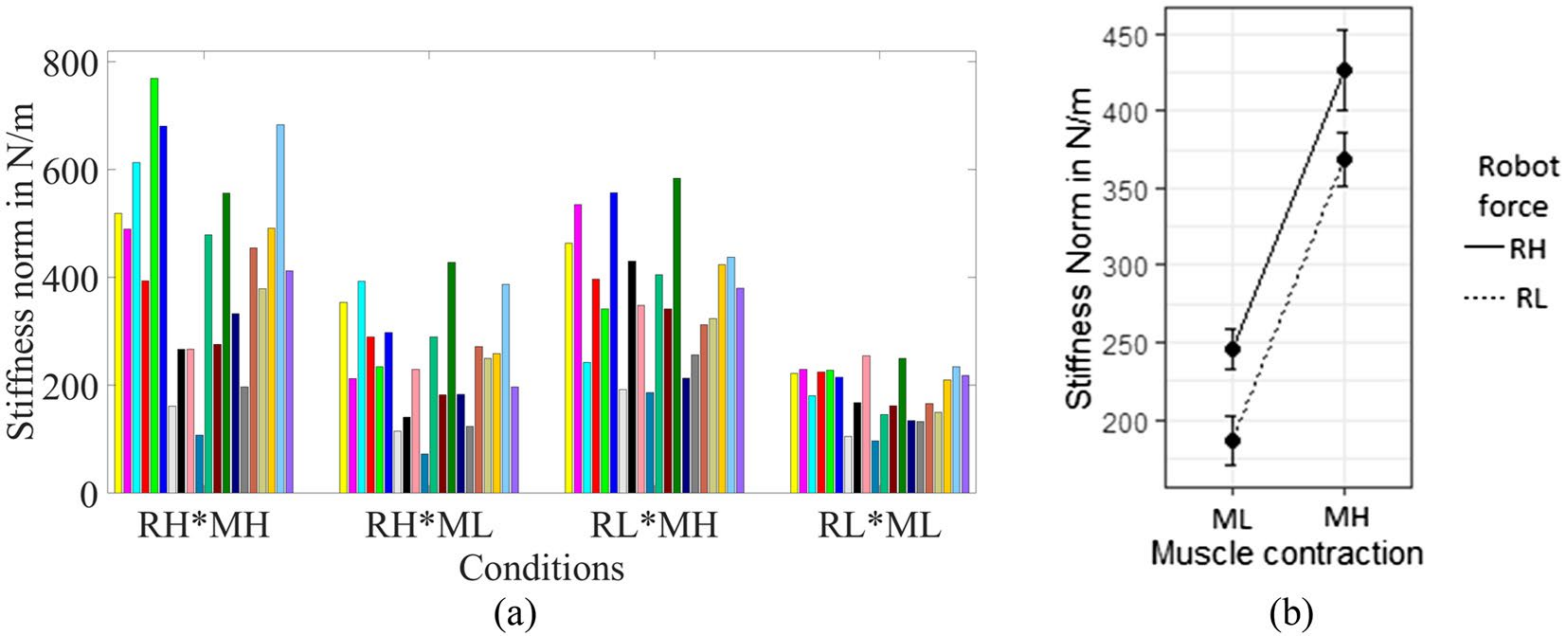
Norm of the arm stiffness increases with the increase of muscle contraction and robot interaction force. The average stiffness was highest during high robot interaction force with high muscle contraction (RH*MH) condition (**a**) experimental results (different colors represent different participants) (**b**) ANOVA analysis of the stiffness norm.

The estimated arm stiffness was dependent on the experimental conditions (Fig. 3a). The norms of the 2 × 2 stiffness matrices computed from the force–displacement relationship after 3 s were averaged across trials and participants for the four conditions. The average arm stiffness norm was the lowest in the RL*ML condition (167.13 N/m) and highest in the RH*MH condition (372.95 N/m), with intermediate values in RH*ML (225.07 N/m) and RL*MH conditions (314.57 N/m). The stiffness was higher in RH than in RL, and in MH than in ML. These trends were statistically significant (*p* < 0.001, Fig. 3b and Table 3). The low robot interaction force (RL) reduced the arm stiffness norm by − 58.47 N/m, whereas the high muscle contraction (MH) increased the arm stiffness norm by 181.56 N/m. The estimated 2 × 2 stiffness matrices are provided in Table 4, which shows the characteristics of typical arm stiffness matrices with low off-diagonal terms^36,45^.

**Table 3:** Fixed effects of stiffness norm using linear mixed model fit by REML and *t* tests use Satterthwaite's method (Stiffness ~ Robot.force + Muscle.contraction + (1 | Participant)).

	Estimate	Std. Error	df (degree of freedom)	t value	Pr( >|t|)
Intercept	245.26	25.26	34.44	9.71	*p* < 0.001***
RL	− 58.47	18.70	58.00	− 3.13	0.003**
MH	181.56	18.70	58.00	9.71	*p* < 0.001***

*p < 0.05, **p < 0.01, ***p < 0.001.

**Table 4:** Estimated average stiffness values of all 20 participants.

Stiffness (N/m)	Conditions			
	RH*MH	RH*ML	RL*MH	RL*ML
K_xx_	312.81	205.11	263.67	156.11
K_xz_	− 8.36	−17.13	17.72	− 8.02
K_zx_	21.86	− 20.67	26.30	− 10.93
K_zz_	371.85	207.15	305.01	158.98

## Discussion

Forces applied to the hand may be sensed by the respective force sensors at the hand, such as the cutaneous pressure receptors on the palm. However, when the pressure on the palm is high due to a strong hand grip, the cutaneous pressure sensors on the skin may suffer from decreased sensitivity to small changes in the pressure, since our ability to detect a change in pressure (the Just Noticeable Difference, or JND) tends to be about 8-10% of the current stimulus intensity. This makes the detection of small interaction forces to be less effective through the pressure sensors on the hands in our MH conditions where the co-contraction of the forearm muscles increases the grip force. Indeed, the approximate grip force for the high muscle contraction (70-80% MVC) was 20–30 N^36,46–48^, meaning that a 1 N applied force is an increase of only 3-5%, likely below the threshold for detection. In contrast, during low muscle contraction (0~20% MVC) grip force was less than 5 N, so 1 N of applied force should be at least a 20% change.

However, detecting a change in cutaneous pressure is not the only way to sense the applied force. As force is applied to the hand, the joints in the arms are displaced as a result, unless the human body is completely rigid which is impossible. This displacement is picked up by the proprioception on muscles and tendons (ex. the Golgi-tendon organs and/or the muscle spindle), which are known to sense kinematics. That is, forces at the hands may be sensed as the arm is displaced as a result, regardless of whether the applied force is above the detection threshold of the cutaneous pressure receptors. To our knowledge, this work is the first to provide evidence for this alternative force sensing through proprioception. This is especially relevant in scenarios in which the handgrip must be tight to ensure the security of the mechanical coupling between the two partners (ex. providing balance assistance during walking). Indeed, our experiment suggests that the participants may be utilizing these kinematic sensors as an effective force sensor. It was observed that the sensitivity of the interaction force direction was higher when the radial displacement/movement of human arms were larger. In addition, the sensitivity of the interaction force was higher when the muscle contraction was low that reduced the pressure on the palm. In this view, participants may have sensed the direction of the interaction force by sensing the displacement/movement of their arms and/or from the changes in the pressure on their palm when the grip was not tight (ML conditions). When the grip was very tight (MH), however, the hand-robot handle coupling between the human and the robot served mainly as a mechanical connection that allowed the interaction force to generate arm displacements which are eventually sensed by the proprioceptors. This view is consistent with the recent observation that the muscle spindles may encode forces during stretch^49^. Although there are two parallel sources of information for force sensitivity (cutaneous force sensor and proprioception), this experiment was designed to emphasize a scenario where the mechanoreceptors on the skin may not be effective due to tight handgrip and thus proprioception would be more important (ex. overground physical interaction tasks). However, various other scenarios could exist where the relative importance of these channels would vary (or even negatively interfere). To further investigate this, future experiments may alter the sensations through the two channels. For example, the arm could be fixed to oblige the participants to use the cutaneous pressure receptors on the palm, allow softer grip, provide steeper changes in the interaction forces, or allow self-selection of arm postures (ex.^44^) to study the effect of joint configurations to force sensitivity.

In this experiment, all participants were asked to sense the direction of interaction force through the senses in their arms and hands without any visual feedback. In all four combinations of conditions, the participant’s arms were displaced from the initial position in X and Z-directions due to the applied interaction force from the robot handle. Participants were stiffer in the Z-direction (*K*_*zz*_ > *K*_*xx*_) than X-direction and correspondingly, the sensitivity was lower in the Z-directions (+Z, −Z) than X-directions (+X, −X). The observation that the stiffness in Z direction was higher than in the X direction is consistent with prior findings^45^. Participants were also more sensitive in the positive directions (+X, +Z) than in the negative directions (-X, −Z). While this effect is significant, the biomechanical basis for this observation is unknown. It was observed that human arm displacement was higher during low muscle contraction conditions (ML) in which the arms are estimated to be less stiff than in the high muscle contraction conditions (MH) as shown in Fig. 3b. This implies a relationship between the arm stiffness and the force sensitivity^50^. The arm displacement was the smallest in the low robot interaction force and high muscle contraction (RL*MH) condition. The movement of human arms increased at a higher robot interaction force than low robot interaction force for the same level of muscle contraction. This observation is analogous to the results in^51^ where bigger force is easier to discriminate and promote haptic communication between humans.

On the other hand, participants' ability to sense the interaction force direction was high when the arm displacement was also high - which occurred when the estimated arm stiffness was low. For example, the sensitivity of the direction of interaction force was higher in the RL*ML condition than the RL*MH condition. Hence, with the same level of robot interaction force in the same specific posture of the human arm, the sensitivity of the interaction force direction varied depending on the level of muscle contraction/stiffness/muscle activation level of the human arm. In addition, for the same specific posture, experimental trials where the arm was stiffer (less displacement) were less likely to be correct than trials with low arm stiffness (high displacement). Overall, human arm movement was related to the correctness of the interaction force/sensitivity of the interaction force direction. Hence, humans may benefit from lowering their arm stiffness as it would help them to increase the displacement of the arm, allowing even small interaction forces to be detected.

The estimated magnitudes (norms) of the 2 × 2 stiffness matrices are smaller when the robot force was low (RL) and higher in RH, despite the fact that the muscle contraction levels were kept similar, as shown in Fig. 3b and Table 3 (*p* < 0.003). A possible explanation for this phenomenon is that it is due to the well-known nonlinear force-displacement relationship of skeletal muscles. Given a nonlinear force-displacement curve originating from a specific level of muscle contraction and posture, the slope (stiffness) of the curve is high for high robot interaction force and low for lower robot interaction force. As a consequence, a linear approximation of the arm stiffness would be lower with low robot interaction force. That is, even if the participants did not modulate their muscle contraction level (%MVC), the arm stiffness may be estimated differently depending on the applied force level.

Nonetheless, there still is a possibility of voluntary modulations of the muscle contraction by the participant, due to the inherently variable muscle activity recordings that cannot completely rule out such cases. In this regard, a possible alternative explanation to the lower stiffness in RL conditions is that there may be an unmeasured lowering of muscle contraction in RL conditions, intentionally or otherwise, so as to be more sensitive to the low level of interaction force. This lowering of the arm stiffness may not have occurred as prominently in the RH conditions since the higher interaction forces are easier to detect even without lowering the arm stiffness to take advantage of the proprioception.

However, it is emphasized once again that a direct measure of the interaction force was not available in this research, and thus the reported arm stiffness is not a direct measurement. Therefore, further research is required to find the variation of endpoint stiffness with different levels of the interaction force.

Better pHRI may be possible by lowering the stiffness of the robot arm to mimic the characteristics of the human arms. Effective pHRI begins from a better understanding of how human participants communicate movement intentions with their partners through the physical coupling. It has been suggested that humans can effectively guide other humans by hand by using interaction forces to communicate intentions during walking^16,19,20^,handshaking^17,18^, etc. In this work, it was suggested that humans are more sensitive to the interaction forces when their arm stiffness is lower. Humans may expect their partner’s arm stiffness to be lower because it is natural and advantageous for them to communicate through interaction forces. If so, in pHRI, the humans may also expect their robot partners to have a compliant, low-stiffness arm, rather than a stiff and sluggish arm. A low-stiffness robot arm may be regarded as more human-like. Additionally, compliant participants (low arm stiffness) were more sensitive than stiffer participants (high arm stiffness) for the same level of interaction forces (RH or RL)^50^.Therefore, it could be speculated that this is at the basis of reduced sensitivity in participants with a pathologically high level of limb stiffness, as for spastic patients.

There are a number of valuable additional benefits of a low-stiffness robot arm. For example, a soft, easy-to-manipulate robot arm is less likely to be a safety threat to a human partner^52,53^ and may help the subjective quality of the pHRI to improve. This may be especially important in healthcare applications where a robot may interact with frail populations. Also, to provide low stiffness, a robot arm may be designed using smaller actuators or power sources to reduce development cost and the overall size of the robot.

Note, however, that robots do not require low stiffness for increased force sensitivity. Their sensors (electromechanical transducers) do not suffer from the same reduced sensitivity at a higher force that is common in human perception. Hence, if all the robot needs is to sense the interaction force from the human partner, its arm impedance is irrelevant. The low arm stiffness of the robot would be for the benefit of the human partner, and not as much for itself. However, the role of arm stiffness is not restricted to a single goal. It is well known that humans increase their arm stiffness to improve stability^45,54^. For tasks that require both high sensitivity to interaction forces (low stiffness) as well as good stability (high stiffness), sophisticated modulation of arm stiffness may be required. The central nervous system (CNS) may make swiftly switching decisions on when to stiffen or loosen an arm, depending on the state of the interaction at that moment. Neither consistently high nor low arm stiffness may be ideal for any given scenario, which warrants further research on arm stiffness modulation on complex tasks.

This work was mainly inspired by the necessity to implement intuitive and effective pHRI. It is suggested that low muscle contraction may help increase the sensitivity to the small interaction forces, which may contain movement intentions of the partner, by allowing higher arm displacements to occur. The results of this work imply that the lower robot arm stiffness or human arm muscle contraction may be the desired characteristics of pHRI and pHHI. The findings of this experiment can be used to guide the design of a robot for physical interaction tasks with a human.

## Materials and methods

### Experimental setup

20 healthy young adults (19 males and 1 female), 18 to 35 years of age (22.1 ± 4.025 years) without a self-reported history of neuromuscular injuries or disorders participated in this study. All participants reported themselves to be right-handed. The experimental protocol and procedure were approved and in accordance with relevant guidelines and regulations of the institutional review board (IRB) of the University of Missouri. All participants gave their written informed consent and were free to withdraw their participation at any time. The hypothesis and the experiment design are preregistered in the open science foundation (OSF: osf.io/qr785).

**Figure 4:**
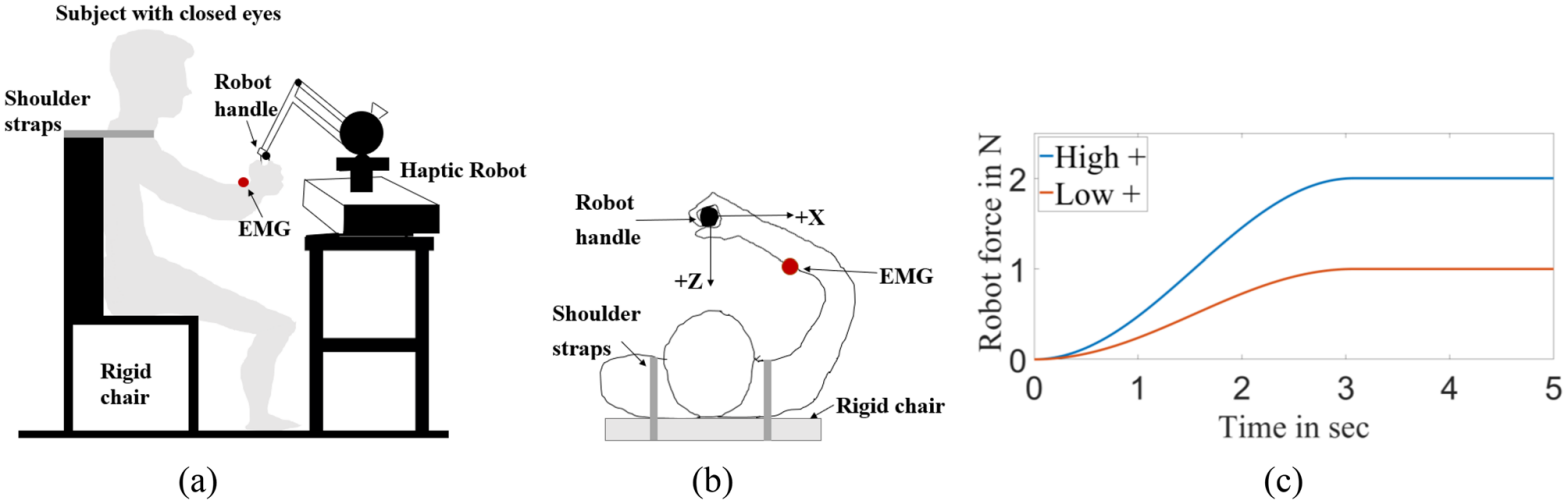
Seated human posture during the experiment with a haptic robot (**a**) experimental setup (**b**) top view of the experimental setup (**c**) applied robot interaction force profile for a trial of ~ 5 s.

The experiment involved externally applied interaction forces to the hand of a seated participant as he/she relaxed or contracted their lower arm muscles. All participants were seated in a rigid chair to keep their back against the chair at all times. Shoulder straps were used to help maintain their posture as depicted in Fig. 4a,b ^55^. Using their right hand, participants grabbed the handle of a haptic robot (Phantom Premium 1.5/6 DOF-HF, 3D Systems, Rock Hill, SC, USA) in front of them as shown in Fig. 4b. The right arm was posed such that the distance from their sternum to the right hand was approximately 30% of their arm length, with the shoulder abduction angle of ~71°, shoulder horizontal flexion of ~45°, elbow flexion angle of ~ 90°, and the forearm and wrist in its neutral position (~0°)^55,56^. The strength of the grip was inferred by the level of activity of the hand-grip muscles on the forearm^46,57–59^ using single-channel electromyography (Muscle SpikerShield Bundle model #V2.61, Backyard Brains, Inc. MI, USA) above the forearm flexor muscle group. A high grip force was identified as 70–80% of the maximum voluntary contraction (MVC) of the forearm muscles, whereas a Low grip force was identified as 0~20% of MVC in such a way that there was a clear distinction between high and low grip forces.

Because the participants were asked to maintain their posture at all times, the contraction of the forearm flexor muscle groups was accompanied by a co-contraction of the whole forearm muscles. A haptic robot was used to apply interaction force in two different magnitudes in one of four directions to a seated participant as shown in Fig. 4. The robot applied a force that gradually increased from 0 → 1 N (Low) or 0 → 2 N (High) over a 5-second duration in such a way that the maximum level of interaction force was reached ~3 s (Fig. 4c). The slow increase in the force was to avoid stretch reflex^56^. After ~3 s the level of interaction force remained maximum constant value (2N or 1N) until ~5 s when a single trial was ended as presented in Fig. 4c. Then, the level of interaction force remained constant until 5 s. The robot provided this force to the hand in one of the four directions on a horizontal plane (+X, −X, +Z, or −Z directions, Fig. 4b). The direction of the interaction force was the target information that the robot provided to the human, whereas the level of the interaction force was the intensity of that information. The magnitude of the force was controlled in an open-loop manner where the appropriate motor torque profiles were commanded to the robot by the experimenter. The position of the robot handle (which is also the position of the participant’s hand) was measured by the encoders of the robot joints. The resolution of the handle position sensing at the configuration shown in Fig. 4a was ~10^−6^ m. There was no dead zone in the system.

### Experimental protocol

The aim of this study was to find what affects the sensitivity of the interaction force during physical interactions in humans. In this experiment, participants were asked to identify if the direction of push as the robot provided the interaction force at the hands. Participants were blindfolded to encourage them to focus on the sensation at their hands to identify the direction of the push.

At each trial, the robot-provided interaction force was either high (2 N) or low (1 N), and the grip on the robot handle was either high (70~80% of MVC) or low (0~20% of MVC). It can be considered a low robot interaction force as RL, high robot interaction force as RH, low muscle contraction as ML, and high muscle contraction as MH. Therefore, there were four different conditions in the experiment such as high robot interaction force with high muscle contraction (RH*MH), high robot interaction force with low muscle contraction (RH*ML), low robot interaction force with high muscle contraction (RL*MH), and low robot interaction force with low muscle contraction (RL*ML).

Participants had no knowledge of the intensity setting of the interaction force in any particular trial. Each time the force was applied, participants were asked to identify whether the direction of the interaction force towards them (+Z), away from them (−Z), to their right (+X), or left (−X), while maintaining their pose (maintained the initial abduction angle, shoulder horizontal flexion angle, elbow flexion angle, and the forearm and wrist position). Indeed, the participant's hand moved in response to the applied robot interaction force. They were allowed to give the response at any time during the 5-second period of the force application. Participants’ responses were recorded as correct, incorrect, or no-response, where they either declared that they could not identify the direction correctly or if they failed to provide a response within 5-s. For each of the four conditions (RH*MH, RH*ML, RL*MH, and RL*ML), there were 6 pushes in each of the four directions (+Z, −Z, +X, and −X), with a total of 96 trials in each experiment session. All 96 trials were equally randomized in the directions and intensity of the interaction forces as well as in the levels of muscle contraction. To avoid muscle fatigue, the randomized sequence of trials was checked to ensure that there were no more than four consecutive high-MVC trials. Also, mandatory ~1 min breaks were provided during the experiment (after each of the four consecutive MH trials). Each trial lasted approximately 10 s. In addition to the correctness of the response, the radial displacement of the hand as a result of the interaction force was recorded throughout the 5-s in all trials.

### Data processing and analysis

For each trial, the measurement included the response (correct, incorrect, or no-response) and the maximum radial displacement

1 $${\text{R}} = \max \left( {\sqrt {{\text{dx}}\left( {\text{t}} \right)^{2} + {\text{dz}}\left( {\text{t}} \right)^{2} } } \right),\quad {\text{t}} = \left[ {0,{ }5} \right]$$

where *dx* and *dz* are the displacements of the handle in the X and Z directions with respect to its initial position at t = 0.

Human arm stiffness was also estimated in the experiment by considering the applied robot interaction force and the resulting hand displacement. While direct measurement of the interaction force was not available, the commanded robot interaction force was used as an approximation of the interaction force value, from which the 2-dimensional endpoint stiffness of the arm was estimated through the following procedure^36,45,60^:

The quasi-static stiffness of the arm is related to the interaction forces and the hand displacement such that

2 $$\left[ {\begin{array}{*{20}c} {{\text{F}}_{{\text{x}}} } \\ {{\text{F}}_{{\text{z}}} } \\ \end{array} } \right] = \left[ {\begin{array}{*{20}c} {{\text{K}}_{{{\text{xx}}}} } & {{\text{K}}_{{{\text{xz}}}} } \\ {{\text{K}}_{{{\text{zx}}}} } & {{\text{K}}_{{{\text{zz}}}} } \\ \end{array} } \right]\left[ {\begin{array}{*{20}c} {{\text{dx}}\left( {\text{t}} \right)} \\ {{\text{dz}}\left( {\text{t}} \right)} \\ \end{array} } \right],\quad {\text{t}} = {3}\;{\text{sec}}$$

where F_x_ and F_z_ are the robot interaction force in the X and Z-direction; *dx* (*t*) and *dz* (*t*) are the displacements in X and Z, respectively, K_xx_, K_xz_, K_zx_, and K_zz_ are the elements of the 2-dimensional stiffness matrix. To avoid dynamic effects, measurements at 3 s were used. Then, the stiffness elements K_xx_, K_xz_, K_zx_, and K_zz_ were determined for each participant for each of the four conditions (RH*MH, RH*ML, RL*MH, and RL*ML) by using the linear least square regression method. However, the magnitude of the velocities of the hand (=robot handle) at ~3 s for all 1960 trials was small (V_x_: 1.39 ± 1.23 mm/sec and V_z_: 1.49 ± 1.21 mm/s). Therefore, we can assume that at ~3 s, the hand is quasi-static and only the stiffness component of the arm dynamics is present at that time.

A two-way measure of analysis of variance (ANOVA) was used to find the effect of the robot interaction force and muscle contractions on the measurement of the sensitivity of the interaction force direction. A generalized linear mixed-effects model was also used to analyze the data in a trial-by-trial manner, where the binomial outcome measure was whether participants responded correctly on that particular trial. For this, no-response was considered as an incorrect response. This analysis included fixed effects of robot interaction force, muscle contraction, motion direction (X/Z and +/−), and the logarithm of the maximum radial displacement on that particular trial, with a random intercept for participant and by-participant random slopes for muscle contraction. The maximum radial displacement was transformed to its logarithmic value due to the skewness and kurtosis of the raw data sets.
